# B-cell non-Hodgkin lymphoma of the ethmoid sinus

**DOI:** 10.5935/1808-8694.20130046

**Published:** 2015-11-02

**Authors:** Paulo Tinoco, José Carlos Oliveira Pereira, Flavia Rodrigues Ferreira, Vânia Lúcia Carrara, Marina Bandoli de Oliveira Tinoco

**Affiliations:** a Specialist on ENT (Coordinator of the Medical Residency Program on ENT at the São José do Avaí Hospital); b Specialist on ENT at the São José do Avaí Hospital (Specialist on ENT at the São José do Avaí Hospital); c Second-year Resident Physician in the ENT service at the São José do Avaí Hospital (Second-year Resident Physician in the ENT service at the São José do Avaí Hospital); d First-year Resident Physician in the ENT service at the São José do Avaí Hospital (First-year Resident Physician in the ENT service at the São José do Avaí Hospital); e Second-year Medical Student (Second-year Medical Student). São José do Avaí Hospital - HSJA

**Keywords:** B-cell, lymphoma, lymphoma, Non-Hodgkin, paranasal sinuses

## INTRODUCTION

Lymphomas are the most common head and neck non-epithelial malignant tumors. They are divided into Hodgkin (HL) and non-Hodgkin lymphomas (NHL). Non-Hodgkin lymphomas account 60% of the cases and are categorized into B-cell (the more common type), T-cell, or NK-T-cell lymphomas^1^.

Some NHLs are extranodal, i.e., they involve tissues other than lymph nodes^2^.

Paranasal sinus lymphomas are rare, have poor prognosis, and their non-specific clinical signs may delay the production of a firm diagnosis^2^.

## CASE REPORT

**Figure 1:**
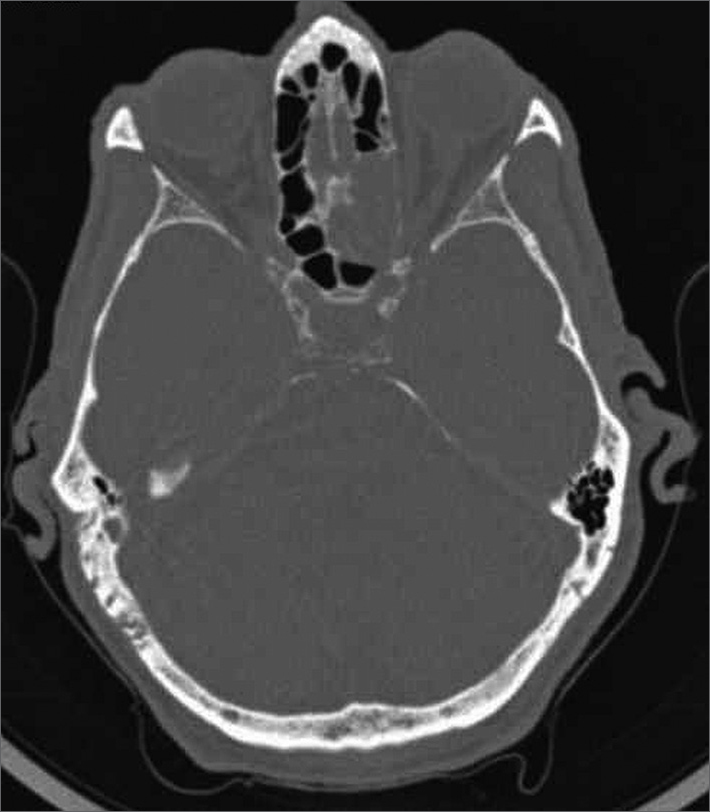
Axial view CT scan: soft-tissue density tumor in left posterior ethmoidal cells measuring 19 × 20 × 30 mm.

A 77-year-old male patient born in Itaperuna, State of Rio de Janeiro, came to our service complaining of dizziness, discomfort, headache, and vomiting that had persisted for 30 days. He had no sinonasal symptoms. The patient underwent routine examination. Skull CT scans were ordered because of the symptoms he manifested, and a tumor with soft-tissue density measuring 19 × 20 × 30 mm was seen in the left posterior ethmoidal cells, in addition to erosion of the lamina papyracea (Figure 1). A rigid 0° scope was used to biopsy the patient. However, as the ethmoidectomy was performed, the surgeon felt the tumor could be easily resected. The tumor was removed and sent to the pathologist for analysis. The report came back categorizing it as lymphoproliferative disease, and immunohistochemistry found it to be an aggressive, CD 20-positive (marker for B-cells), CD 45-positive (marker for lymphocytes) B-cell non-Hodgkin lymphoma with necrotic areas.

The patient was referred to oncology care and offered cycles of COP (cyclophosphamide, vincristine, and prednisone) along with confor-mational radiotherapy with a total dose of 4140 cGy/23 fractions. He responded well to the treatment. Control contrast-enhanced CT scans taken six months after surgery did not reveal remaining disease or metastasis. However, the patient later developed severe interstitial pneumonia induced by cyclophosphamide toxicity and died.

## DISCUSSION

Non-Hodgkin lymphomas rank second in prevalence among malignant neoplasms of the head and neck. They may have different cell phenotypes^1^ and are categorized as indolent, aggressive, and highly aggressive^2^.

The patient had diffuse large B-cell lymphoma - the most common subtype of NHL. This type of lymphoma accounts for 30% of NHL cases and usually involves males aged between 40 and 70 years. Diffuse large B-cell lymphomas are often aggressive, and include fast node involvement and extranodal disease in up to 40% of the cases^3^.

Approximately a third of the cases of NHL include extranodal disease and involvement of the gastrointestinal tract, the skin, and the oral mucosa. Paranasal sinus lymphomas are seen in 9% to 13% of the cases, mainly in B-cell lymphomas, and involve the maxillary and ethmoid sinuses^4^.

Symptoms are generally non-specific, and may include repetition rhinosinusitis, nasal obstruction, pain, and facial edema^4^. B symptoms (fever > 38° C, nocturnal sweating, and 10% weight loss in six months) occur in 40% to 45% of the cases, and even more frequently in aggressive disease^5^. As the disease evolves, necrosis and bone lysis set in, producing fetid odors.

Diagnosis is performed through meticulous physical examination of the head and neck and imaging. Histopathological testing of the tumor confirms the diagnosis of lymphoma and immunophenotyping differentiates primary nasal lymphoma - derived from T-cells - from B-cell nasopharyngeal lymphoma (69%)^4^, ^5^.

According to the Ann Arbor staging system (also used for HL), our patient was on stage IEA, as only one extranodal structure was involved (ethmoid sinus) and he had no B symptoms, thus excluding other possible primary involvement sites^5^.

Treatment is based on surgery, systemic chemotherapy, and localized radiotherapy, and patient mean survival is five years. Regional and distal metastases are rare^4^, ^5^.

## CONCLUSION

Lymphomas are the most frequent non-epithelial malignant tumors of the nose, and must be included in the differential diagnosis of paranasal sinus neoplasms. Proper workup and definitive diagnosis are fundamental for the early introduction of treatment and improved survival of NHL patients.
